# Small Non-Coding RNAs in the Human Placenta: Regulatory Roles and Clinical Utility

**DOI:** 10.3389/fgene.2022.868598

**Published:** 2022-03-30

**Authors:** Nikita Telkar, Greg L. Stewart, Michelle E. Pewarchuk, David E. Cohn, Wendy P. Robinson, Wan L. Lam

**Affiliations:** ^1^ British Columbia Children’s Hospital Research Institute, Vancouver, BC, Canada; ^2^ Department of Medical Genetics, University of British Columbia, Vancouver, BC, Canada; ^3^ British Columbia Cancer Research Centre, Vancouver, BC, Canada

**Keywords:** placenta, small non-coding RNA (sncRNA), epigenetics, gene regulaiton, pregnancy

## Abstract

The placenta is a vital organ formed during pregnancy, and being the interface between the mother and fetus, it is paramount that placental functioning is strictly controlled. Gene expression in the placenta is finely tuned—with aberrant expression causing placental pathologies and inducing stress on both mother and fetus. Gene regulation is brought upon by several mechanisms, and small non-coding RNAs (sncRNAs) have recently been appreciated for their contribution in gene repression. Their dysregulation has been implicated in a range of somatic and inherited disorders, highlighting their importance in maintaining healthy organ function. Their specific roles within the placenta, however, are not well understood, and require further exploration. To this end, we summarize the mechanisms of microRNAs (miRNAs), Piwi-interacting RNAs (piRNAs), small nuclear RNAs (snRNAs), small nucleolar RNAs (snoRNAs), and transfer RNAs (tRNAs), their known contributions to human placental health and disease, the relevance of sncRNAs as promising biomarkers throughout pregnancy, and the current challenges faced by placental sncRNA studies.

## Introduction

The human placenta is the temporary, yet essential, organ responsible for the progression of a healthy pregnancy and of fetal development. Although it has many important functions, the predominant role of the placenta is to facilitate all nutrient, gas, and waste exchange between the mother and fetus. Impaired placental function can disrupt this interchange between the two, causing severe fetal and maternal distress (Ray et al., 2005). Accordingly, gene expression in the placenta is firmly regulated at every stage of pregnancy, however, inherent biological characteristics such as gestational age, sex of the fetus, and maternal health can influence this expression (Sood et al., 2006). Furthermore, different placental cell types display their own unique expression profiles, which may differ from that of the aggregate placental tissue (Pique-Regi et al., 2019). Expression changes are also observed in placental pathologies (Nishizawa et al., 2011), which may cause long-term repercussions on the health of both affected mothers and fetuses, including higher predisposition to cardiovascular, respiratory, metabolic and psychiatric diseases (Thornburg et al., 2016). Early placental functioning has also been likened to that of cancer progression (Costanzo et al., 2018). Despite the far-reaching impacts of the placenta throughout birth and into adulthood, much of its function and subsequent impacts remains understudied, and the factors governing placental gene regulation even more so. While animal models have provided us with foundational workings of the mammalian placenta, they do not recapitulate the molecular, biological, and genomic makeup of the human placenta (Hemberger, Hanna, and Dean 2020).

Several genetic and epigenetic mechanisms regulate gene expression throughout transcription and translation—the well-known Epidermal Growth Factor Receptor (EGFR) expression feedback loops (Segatto, Anastasi, and Alemà 2011); hypermethylation in gene promoters which repress gene expression (Yang et al., 2014); histone modifications, such as H3K27me3 which causes repression, and H3K27ac which promotes activation (Lavarone, Barbieri, and Pasini 2019). Non-coding RNAs (ncRNAs)—RNA molecules that do not translate into a protein—have recently garnered attention in their role as gene regulators (Cox et al., 2015). While several long ncRNAs (lncRNAs) are known to be involved in placental gene regulation (such as the oncofetal H19 lncRNA (Raveh et al., 2015)), the contribution of small ncRNAs (sncRNAs) is less understood and represents an active area of exploration (S. Gong et al., 2021). Their stable, double-stranded structure and small size (Supplementary Table 1) allows these molecules to cross the placental barrier and be released into maternal circulation (Bai et al., 2021), and thus, they may have considerable potential as measurable biomarkers throughout pregnancy. These sncRNAs could prove to be prime candidates for early pregnancy diagnostics, and possibly even for the prophylactic care of pregnant people.

Here, we highlight the known roles of sncRNAs in human placental biology, the limitations that currently exist in placental studies, the importance of developing novel methodologies for understudied sncRNA species, and why sncRNAs should and can be considered as potential placental and pregnancy diagnostic biomarkers.

## SncRNA Expression Impacts Placental Function

SncRNAs are post-transcriptional regulatory molecules that comprise several distinct species (Esteller 2011). They range from 20–200 nucleotides and predominantly downregulate gene expression by either degrading or altering messenger RNA (mRNA), or by modifying ribosomal RNA (rRNA). The role of sncRNAs has been extensively researched in various cancer subtypes, with several studies showing their expression levels to be altered in malignant tissue (Ferreira and Esteller 2018). SncRNA transcriptome profiling of placentas ranging from early gestation to term revealed that microRNAs (miRNAs) were the most abundant sncRNA species, followed by Piwi-interacting RNAs (piRNAs), small nucleolar RNAs (snoRNAs), small nuclear RNAs (snRNAs), and finally transfer RNAs (tRNAs) (Martinez et al., 2021a). SncRNA species are potential disease biomarkers or clinically actionable targets—for example, miRNA hsa-miR-16 can directly downregulate the *VEGF* gene, which is vital in establishing and maintaining the placental vasculature, by binding specifically to its 3’ untranslated region (UTR) (Zhu et al., 2016). Furthermore, overexpression of hsa-miR-16 has been observed in women with recurrent spontaneous abortions, and *in vivo* mouse studies have demonstrated that overexpression of hsa-miR-16 is associated with a reduction in placental and offspring birth weight (Maccani, Padbury, and Marsit 2011). Additionally, aberrant expression levels of hsa-miR-16, which acts as a tumour suppressor, and its target genes have been reported in several cancers (Rivas et al., 2012). Determining the role of sncRNAs in the genetic regulation of the placenta is critical to understanding the workings of this crucial organ.

### miRNAs Regulate the Placental Transcriptome Dynamically

**FIGURE 1:**
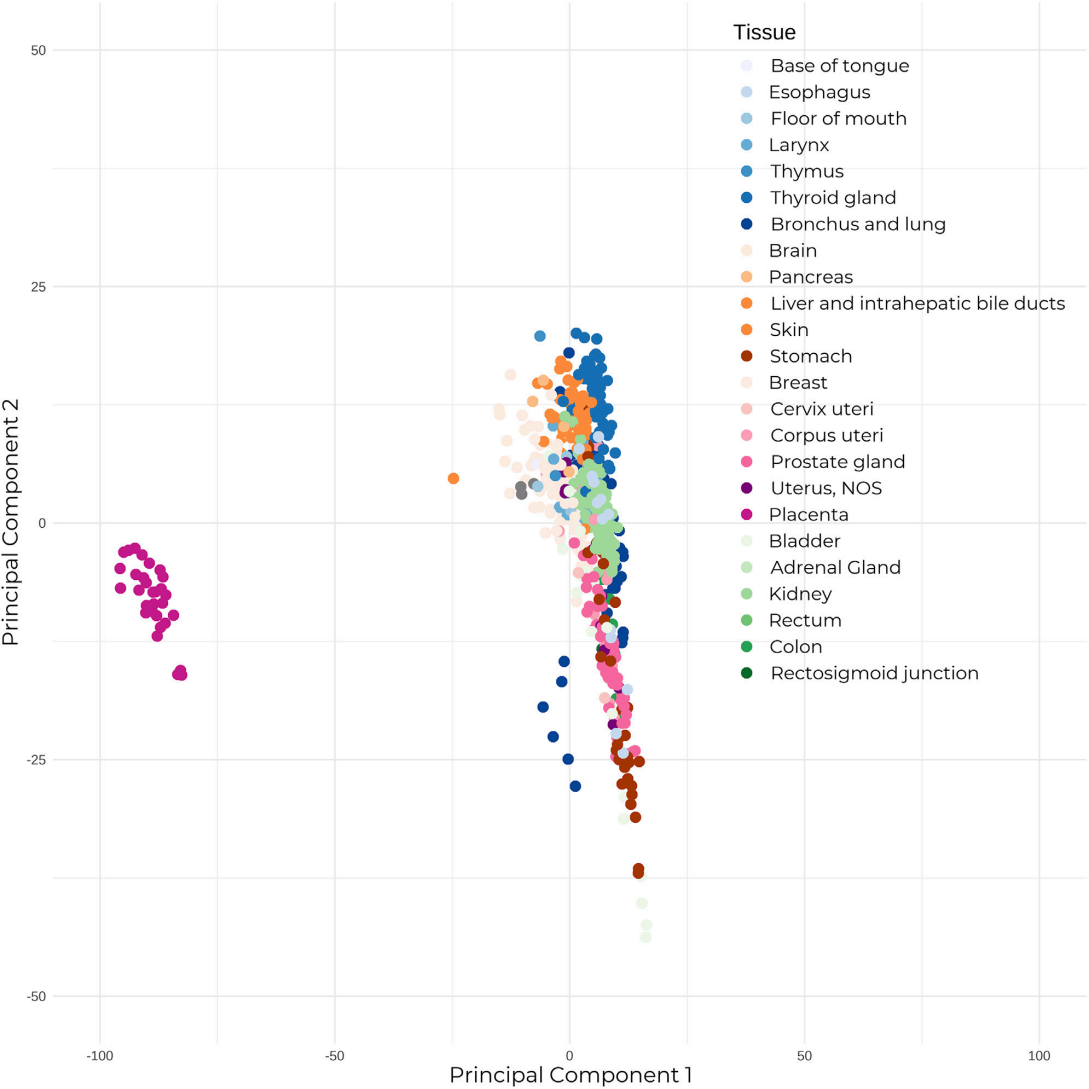
The placenta shows significantly different expression to adult healthy tissue. Mature microRNA expression between 30 placental tissue samples, and 23 healthy adult human tissue (679 total samples). Expression counts were TPM-normalized, and samples were subjected to Principal Component Analysis. Placental samples annotated in dark pink on the left show clear segregation from the adult tissue samples.

The majority of placental studies have focused on miRNAs, which can be transcribed from both genic and intergenic regions, and are usually present in clusters. After being loaded into the RNA-induced silencing complex (RISC), the seed sequence (nucleotides 2–8) of miRNAs binds with perfect complementarity to mRNAs, while the remaining miRNA sequence binds with partial complementarity—allowing one miRNA to target several mRNA transcripts due to this non-specific binding, and thus deregulating their expression. Unsurprisingly, these species exert significant regulatory effects on the genome. Short hairpin RNAs (shRNAs) have the same biogenesis and function as miRNAs, with the exception that where miRNAs show imperfect binding within their looped single-stranded structure, shRNAs show perfect nucleotide binding. When compared to adult healthy human tissue, placental miRNAs show a clearly distinguished expression profile (Figure 1). Placentally-expressed imprinted clusters of miRNA loci have been identified on chromosomes 14 and 19 (Cox et al., 2015), and the miRNAs belonging to these two clusters (C14MC and C19MC) show significantly higher placental expression than the rest of the genome (Gu et al., 2013). The C14MC miRNAs are expressed from the maternally inherited gene copies and show an inverse relationship with gestational age, with the highest expression in the first trimester (Morales-Prieto et al., 2012). Conversely, the placental-exclusive C19MC miRNAs expressed from the paternal allele display the reverse trend, showing increased expression as pregnancy progresses. Several miRNAs are specific to placental pathologies; for example, hsa-miR-210, which is influenced by the expression of the *HIF-1⍺* hypoxia response gene, shows increased expression in preeclamptic placentas when compared to controls (A. Chen et al., 2021; Sun et al., 2021; Frazier et al., 2020). Moreover, recent studies have revealed miRNA isoforms (isomiRs) that have variations in their mature miRNA sequences, which further expands the repertoire of mRNAs targeted for regulation.

Target genes of these placental miRNAs exhibit enrichment for angiogenic, proliferative and immunomodulatory properties. Pathway enrichment analyses reveal these genes to be concentrated within signaling pathways such as EGFR, WNT, and mTOR—pathways are that are also known to be altered in cancer progression. First trimester miRNAs show enrichment for genes involved in cell invasion and proliferation, while third trimester miRNAs are mostly involved in immune regulation. Differential expression by other variables such as sex and ancestry have also been described, however only few miRNAs have consistently shown a difference in expression deemed to be biologically relevant (Hughes et al., 2015).

### piRNAs Exhibit a Placental-Signature Profile

piRNAs are the most numerous small RNA species, and function by binding to PIWI proteins and forming the piRISC. They predominantly repress expression of repetitive DNA, such as transposable elements (TEs), by recruiting DNA methylation machinery in germline cells and tissues. However, there is also evidence of piRNAs adopting a miRNA-like mechanism and causing mRNA decay (Ng et al., 2016). PiRNAs are transcribed through a variety of mechanisms—from specific piRNA clusters, mRNAs, lncRNAs, and, most strikingly, through self and anti-sense piRNA transcripts (*via* the ping-pong cycle). They are evolutionarily conserved in mammalian placentas (Chirn et al., 2015), and failure of expression in germ cells leads to infertility (Hong et al., 2016). Some piRNAs are predominantly expressed in the placenta—for example, the *MEG8* gene in the imprinted DLK-DIO3 cluster (Martinez et al., 2021b) within the C14MC locus harbours some of the highest expressed placental piRNAs. Knockout of piRNAs in mouse sperm cells has been associated with increased expression of TEs and LINE-1 elements, and piRNA expression is critical for mouse spermatogenesis. Overexpression of piRNAs can depress cell invasion and proliferation phenotypes, suggesting a potential role for piRNAs in the pathogenesis of disorders of placental invasion, and require more dedicated profiling in the placenta.

### Small Nuclear RNAs are Differentially Expressed in Placental Cell Types

SnRNAs play a role in pre-mRNA processing, and consist of the spliceosomal U RNAs and the small Cajal body-specific RNAs (scaRNA) subtype (Karijolich and Yi-Tao, 2010). SnRNAs primarily function in small ribonucleoprotein complexes (snRNPs) to direct intron splicing. The only placental-snRNA study in the literature (Goldman-Wohl et al., 2013) shows that the U2 and U6 snRNAs were present at reduced expression levels in synctiotrophoblast versus cytotrophoblast cells. However, upon fusion of cytotrophoblasts into the synctiotrophoblast, the expression dropped ∼3-folds. These species have demonstrated roles in tumour development, where mutations in the U1 snRNA lead to abnormal splicing and inactivation of the tumour suppressor gene *PTCH1* and activation of the *GLI2* oncogene (Suzuki et al., 2019). These mutations were present in 50% of medulloblastomas, with disruptions in the Sonic Hedgehog signaling pathway. The above examples highlight the importance of snRNAs in developmental biology.

### Small Nucleolar RNAs in Pregnancy and Developmental Complications

SnoRNAs, sometimes considered a subtype of snRNAs, are usually present in introns as clusters. They chemically modify rRNAs and some snRNAs, altering protein expression, and also seem to synthesize other sncRNA species, deriving miRNA-like and piRNA-like snoRNAs. SnoRNAs carry out two distinct functions depending upon the motif they possess: either DNA methylation by the C/D box motif, or pseudouridylation via the H/ACA box (Liang et al., 2019). Of these snoRNAs, SNORD115 was found to regulate the alternative splicing of pre-mRNAs as well as of SNORD116, which also targets pre-mRNA splicing. Mutations in the proteins that interact with H/ACA box itself (dyskerin, NHP2 and NOP10) have been associated with X-linked dyskeratosis congenita, a bone-marrow disorder in pregnancy complications (Giri et al., 2018).

SnoRNAs have been observed to predominantly affect neuronal genes, and show differential expression between normal and malignant tissues of other organs (J. Gong et al., 2017), although no studies have investigated human placenta-specific snoRNAs.

### Transfer RNAs are Highly Impactful Gene Regulators

Transfer RNA-derived small RNAs (tsRNAs) arise from the cleavage of tRNAs, and are divided into tRNA-derived stress-induced RNAs (tiRNAs) and tRNA-derived fragments (tRFs) processed from mature and pre-tRNAs (Su et al., 2020). TsRNAs appear to bind mRNAs in a miRNA-like manner to dysregulate target gene expression, while also enhancing RNAi functionality by binding to the AGO family of proteins (Kumar et al., 2014), and have additionally been shown to silence long terminal repeat retrotransposons (Schorn et al., 2017).

Neurodevelopmental pathways have been the chief focus to study tsRNAs, and a study found that introducing sperm tsRNAs from high-fat diet male mice into normal zygotes led to altered gene expression and metabolic profiles in the offspring (Q. Chen et al., 2016)—demonstrating their trans-generational effects. Currently, only one study (Su et al., 2020) has investigated these species in the placenta. TRFs in the mouse placenta were observed to be longer, with a higher number of species of nucleus-derived tsRNAs present in the placenta than the fetal brain or liver, with the expression of tRNAs changing with progression of pregnancy from gestational age E12.5 to E18.5. Immune response induction in these mice showed a change in the tRNA expression profile initially in the placenta, but stabilized over time. This work suggests that tRNA species play a role in the placenta, but their functioning is yet to be determined.

While many types of sncRNAs have been established as crucial players in gene regulation, the majority of sncRNA studies in the placenta have focused on miRNAs. A deeper characterization of the species and their functional targets is needed to improve our understanding of how this unique and indispensable organ functions.

## SncRNAs in Detecting Placental Disorders

The primary options available for the detection and quantification of sncRNAs are microarrays, RNA-sequencing and RTqPCR, which are usually performed in research settings. Currently, routine tests in pregnancy include non-invasive prenatal testing which include measuring serum protein levels of human chorionic gonadotropin and alpha-fetoprotein to screen for common chromosomal abnormalities. These protein tests, however, are usually administered in the second trimester, and recent studies have suggested additional roles of these proteins in the detection of preeclampsia (PE) and gestational diabetes (Amini et al., 2020). As such, expression profiling of specific sncRNAs earlier during screening may prove to have improved benefits for both doctor and mother.

A number of placental studies have observed miRNAs that show differing expression in the placenta with gestational age, PE, and in placentas of intrauterine growth restriction (IUGR) fetuses (Mayor-Lynn et al., 2011). PE is characterized by shallow invasion of placental trophoblasts (TB) into the maternal uterus—the degree of invasion of these TBs is precisely controlled, wherein on reaching the maternal myometrium, these TBs terminate into placental bed giant cells. Thus, genes predominantly participating in cell proliferation and differentiation pathways are often observed to be disrupted (e.g., hsa-miR-21 and hsa-miR-155) in PE (Sun et al., 2021). Placentas from IUGR fetuses, a consequence resulting from a multitude of maternal predispositions, mainly show disturbances in cell-signaling and communication pathways (hsa-miR-675, hsa-miR-27b) (Kochhar et al., 2021; Xu et al., 2021).

Placental sncRNAs, moreover, can diffuse into the maternal serum via exosomes and modulate the maternal metabolic profile, showing rapid clearance after delivery (Luo et al., 2009). As such, the maternal serum miRNA levels from women affected by placental disorders reflects the associated changes in expression observed in the placenta. Extracellular nucleic acids (cell-free DNA/RNA and mitochondrial DNA) which are either free-floating or packaged in vesicles include a host of sncRNAs, and have shown altered blood expression profiles in cancer patients (Schwarzenbach, Hoon, and Pantel 2011), with emerging placental studies observing the same (Hromadnikova 2012; Tsang et al., 2017). Increased presence of these nucleic acids in maternal serum have been observed in pregnancy complications, where expression of interferons and cytokines trigger cellular dysfunction resulting in their diffusion into maternal circulation (Konečná et al., 2018). Several miRNAs originating from extracellular vesicles in maternal plasma were found to differ in expression by gestational age in women with and without gestational diabetes (Nair et al., 2021)—a condition wherein the increased insulin demands required during pregnancy aren’t met.

Furthermore, assessing the protein levels of the genes targeted by these miRNAs in maternal blood, for example, might introduce an early approach in investigating placental and fetal disposition. Efforts for introducing testing for the above described sncRNAs are already underway (Yin et al., 2020), and this sncRNA-specific approach might enable the forgoing of more invasive methods, while also providing a better-informed evaluation of the progression of the pregnancy.

## Overcoming the Challenges in Studying Human Placental sncRNAs

While placental sncRNA-based signatures have great clinical potential, several challenges remain in their development. First and foremost is the variation in techniques utilized to study placental transcriptomics. For example, there are several different methods for processing placental tissue, from the collection of the placental sample to its final analysis. The variation in sample processing times, methods of tissue preservation, and RNA extraction protocols can also introduce discrepancies in the data produced (Mutter et al., 2004; Srinivasan et al., 2019). The profiling method (microarray vs NGS) is a further source of technical variation, and a standardized method does not yet exist (Johnson, Li, and Rabinovic 2007). Moreover, the reference sequences used are not uniform—some studies opt for using precursor miRNA sequences for annotation (MIR369), whereas others utilize the mature miRNA sequences (hsa-miR-369-3p and hsa-miR-369-5p). If the above variations are not accounted for, the expression differences observed may not be biologically relevant, and can lead to inconsistencies between placental studies (Corchete et al., 2020). Placental studies are also generally underpowered due to low sample numbers (Yong and Chan 2020). Furthermore, most pregnancy complications occur during early gestation, and these placental samples remain difficult to obtain, and it also remains unknown whether these rare pre-term samples would have turned pathological were they to progress further in pregnancy. Lastly, accounting for cell composition in whole tissue samples is an essential metric, where expression varies considerably between cell types, but is often overlooked (Yuan et al., 2021).

A solution to low sample size and limited availability of samples would be the promotion of multi-centre collaborations, allowing for increased sample sizes throughout gestation. Other fields have created such consortia to overcome the above issues—the Allen Brain Atlas (Hawrylycz et al., 2012) for neurobiology and cBioPortal for Cancer Genomics (Gao et al., 2013) both house publicly-available data. While one can find publicly-available placental studies on the Gene Expression Omnibus (GEO), not all study data are added to GEO, making it difficult to access and integrate placental data. Building a placental data repository would allow for improving statistical power to achieve more reliable and meaningful interpretations.

Furthermore, this resource and data-sharing would allow for the incorporation of multiple data types, which would provide the ability to identify less frequent alterations that may be missed when looking at gene expression alone. For example, the Placental Growth Factor hormone transcribed from 14q24.3 is regulated by hsa-miR-125b and is overexpressed in PE placentas (Li et al., 2020), while aberrant hypermethylation of this miRNA has been found in hepatocellular carcinoma tumours and cell lines (Alpini et al., 2011). Efforts are being made to improve data-sharing, with a few public databases and applications being recently released that store multi-omic placental and sncRNA data (Table 1), however increasing this effort would exponentially benefit the placental research field.

**TABLE 1:** Online placental and sncRNA resources. Placental databases displaying condition-specific mRNA, sncRNA, single-cell RNA expression. Small non-coding RNA databases include detailed species information, and online tools dedicated to species annotation and expression profiling, exploratory data analysis, correlation and differential expression analyses.

Placental/sncRNA resource	Weblink
POPS Placenta Transcriptome Browser	https://www.obgyn.cam.ac.uk/placentome/
Cellxgene: a scRNA-seq Maternal-Fetal Interface	https://maternal-fetal-interface.cellgeni.sanger.ac.uk/
Placental Methylome Browser: Methylation profiling of CpGs within placental whole tissue and cell types	https://robinsonlab.shinyapps.io/Placental_Methylome_Browser/
Placenta-specific gene sets (Naismitha, K and Cox, B; 2021)	https://ars.els-cdn.com/content/image/1-s2.0-S0143400421001843-mmc1.zip
RNAcentral: The non-coding RNA sequence database	https://rnacentral.org/
miRMaster2: Known and novel sncRNA species annotation and downstream analysis	https://ccb-compute.cs.uni-saarland.de/mirmaster2/
sRNAtoolbox: Expression profiling and prediction of novel sncRNAs	https://arn.ugr.es/srnatoolbox/

## Discussion

The importance of studying the placenta is paramount, but currently not well-recognized. As such, placental studies would significantly benefit from increased data sharing, multi-omics profiling, and collaborative analyses. A focus on exploring the normative placenta is also vital, as only through understanding the functioning of the healthy placenta can we confidently interpret expression profiles of complicated pregnancies and of placental pathologies (miRNAs specific to PE, IUGR-specific, and low-birth specific have already been extensively reported (Yong and Chan 2020)). Assessing sncRNAs which act as biological variable biomarkers would aid in refining the genes or proteins to assess during pregnancy progression. Profiling of these sncRNAs throughout pregnancy within different biological conditions would not only inform us of their role in placental regulation, but also provide for improved care for both fetus and mother during pregnancy.
