# Supplementary material for: Small Non-Coding RNAs in the Human Placenta: Regulatory Roles and Clinical Utility
Source: Front Genet. 2022 Mar 30;13:868598. doi: 10.3389/fgene.2022.868598 (PMC9006164; doi:10.3389/fgene.2022.868598)
Supplement: Supplementary file 1 [file Table1.DOCX]

Supplementary Material

## Supplementary Table 1: Characteristics of small non-coding RNAs. Mode of action, targets, and verified number of species in humans of the common subtypes of small non-coding RNAs.

| **sncRNA** | **Nucleotide Length** | **Targets** | **Complex of action** | **Number of species expressed in humans (database retrieved from)** |
| --- | --- | --- | --- | --- |
| miRNA | 19 - 25 | Several mRNAs | RISC | 1,917 (miRBase) |
| siRNA (ds) | 21 - 25 | One specific mRNA | RISC | Synthetically engineered |
| piRNA | 23 - 36 | TEs, mRNAs | piRISC | 8,438,265 (piRBase) |
| snRNA | 60 - 450 | Pre-mRNA introns | snRNPs | 2,872 (GeneCards) |
| snoRNA | 60 - 300 | rRNAs (maybe mRNA, tRNA, snRNA) | snoRNPs | 2,706 (GeneCards) |
| shRNA | 19 - 25 | Several mRNAs | RISC | Synthetically engineered |
| tRNA | 76 - 90 | mRNAs, TEs | piRISC | 3,202 (ENA) |
